# Agile Development of Polymer Power Transmission Systems for e-Mobility—A Novel Methodology Based on an e-Bike Drive Case Study

**DOI:** 10.3390/polym15010068

**Published:** 2022-12-24

**Authors:** Ivan Demšar, Borut Černe, Jože Tavčar, Nikola Vukašinović, Damijan Zorko

**Affiliations:** 1 Laboratory for Engineering Design LeCAD, Faculty of Mechanical Engineering, University of Ljubljana, Aškerčeva cesta 6, 1000 Ljubljana, Slovenia; 2 Product Development, Department of Design Sciences, Faculty of Engineering—LTH, Lund University, Sölvegatan 26, SE-221 00 Lund, Sweden

**Keywords:** agile development, design, e-bike drive, transmission, polymer gears

## Abstract

The market for electric bicycles has grown extremely and developed rapidly in recent years. To enter such a market with a new product, the development process has to be fast, and throughout the process, feedback from future potential customer(s) should be sought in order to achieve the best possible market acceptance. The article presents the design process of a pedelec e-bike central drive system. The authors were members of the development team and the designers of the mechanical transmission, and therefore had a good overview of the whole project. The development process and the set-up of production require a certain amount of time, during which design changes are inevitable due to changes in customer expectations and demands. The development team should respond to these changes and take them into account during development. Only the ability to react to changes and constant communication with the customer will ultimately lead to a product that can be commercially successful. Based on a critical review of the successfully completed project, general guidelines were established for the development of mechatronic products that consider the principles of Agile methodology. Particular attention was paid to the development of polymer gears, as these were the most demanding components in the system. The presented guidelines were based on an overview of the e-bike R&D process presented, but they can be generalized and used in the development process of any technical physical product. Agile methods were developed in the field of software development and therefore cannot be directly transferred to the field of physical product development. The article highlights and discusses individual special features that distinguish agile development of physical products from software development.

## 1. Introduction

Due to fierce competition, shorter delivery times and product complexity, the biggest challenge in new product development is to increase the innovation and efficiency of the development team to shorten the product development cycle. Recently, agile methods of product development have become established alongside concurrent engineering [1,2,3]. Agile product development (APD), which has already proven itself in software development [4,5,6], is rarely used in the development of physical products.

In the *Agile Manifesto*, Beck et al. [7] presented a software development methodology based on a step-by-step development process divided into small steps that lead to working software code. The process is carried out by small groups and tested at each step. The requirements for this point change depending on the results of the previous steps. As Leite et al. [8] stated, the method stands for maximum adaptability, flexibility of design, time-limited, rapid iterations and a quick response to changing requirements [9,10]. One of the best known ADP methods is Scrum [11], which is intended for solving complex tasks. The Scrum Team (ST), consisting of the development team, the Scrum Master and the Product Owner, forms the core of the method. In his work, Ovsen [12] has shown the possibility of using the Scrum framework and agile methods in the development of new products. Riesener et al. [13] concluded that existing agile methods are not suitable for solving complex physical products.

### 1.1. Blending of Agile and Stage-Gate Methodology at Physical Product Development

Sommer et al. [14] suggest a combination of elements of agile methods and established stage-gate processes. This combination can ensure flexibility, speed and improved communication in the development of new products [15]. Cooper [16] presented a model of how flexible agile elements can be integrated into the stage-gate process and validated the model with two case studies. Agile methods give the stage-gate model powerful tools for micro-planning, day-to-day work control and progress reporting. Agile provides more efficiency and focus, and Stage-Gate enables coordination with other development teams and communications with functions such as marketing and senior management.

Cooper and Sommer [17] report six case studies of large companies experimenting with Agile–Stage–Gate hybrids. The results show that the initial results of these efforts are positive. Some companies report significant improvements in time-to-market and development productivity, as well as faster responses to changing market conditions and customer needs, and higher project team morale. There are challenges in reconciling the two approaches, such as management scepticism and the search for suitable resources for this new model to work. Asmar et al. [18] have proposed a framework for the agile development of innovative product-service systems for physical rehabilitation systems. The planning phase is based on conceptualizing the Minimum Viable Product (MVP), rapid prototyping, and testing functional and business aspects of the prototype. It is important to obtain trustworthy and authentic feedback from potential customers. Gary [19] suggests combining classical and agile project management approaches where they make the most sense. The breakthrough projects that are in the early stages have never been conducted before in the same circumstances. The level of internal uncertainty is high. The team needs creativity, determination and commitment [19].

Companies that use agile stage-gate management (ASGM) can expect shorter development times and a higher degree of innovation, but have to spend more resources to accomplish this. Physical product developers using ASGM experience negative impacts on project resource efficiency due to the need for dedicated resources, frequent product demonstrations, and duplicate management structures [20].

Hendler and Boer [21] argue that digital and physical product development processes have different characteristics and are therefore supported by different approaches. The software development process has to make binding decisions at a late stage in order to use different options. The physical product development process needs to make binding decisions early on, leading to early concept maturity, while later it is often guided by a linear process with high change costs [21]. When a physical and a digital development process are combined, either the former must become more adaptable, the latter less adaptable, or both must change [22]. A combination of measures reduces this problem and leads to optimal performance of the combined process. However, trade-offs are inevitable and require effective coordination and communication between the stakeholders involved [22].

In his research, Smith [23] also introduced the concept of flexible product development, which is largely related to agile development used in software development. At the same time, he proved that the concept of ADP can also be used to increase flexibility and improve performance in product development compared to traditional methods. 

Schuh et al. [24] analysed the agile practical methods and translated them into impact mechanisms. Based on the identified mechanisms of action, agile product development processes can be designed.

Various agile methods are available, hence, a targeted selection of a suitable agile method can be tailored to the company’s boundary conditions and integrated into the company’s existing product development structure to generate hybrid design processes [25]. The concept was applied to four design projects in four different companies. The results showed that methodological support can be useful when choosing an agile method, since project conditions are very different and individual [25]. Schmidt and Paetzold [26] have developed a model to evaluate the maturity level of teams that develop physical products in an agile way from various aspects.

Žužek et al. explored the possibility of introducing Scrum into concurrent product development and proposed a Scrum framework for an agile–concurrent hybrid. Scrum was suggested for carrying out the daily work [27]. The main advantage of the proposed hybrid is that after each iteration, the client reviews the results of an entire loop rather than just one phase, allowing for a broader understanding of the progress and facilitating richer feedback [27].

### 1.2. Prototyping and Agile Methods

Zink et al. [28] argue that prototyping is the key aspect of the agile approach to physical product development. The study sets out criteria for analysing the use of physical prototypes within an agile approach. Reichwein et al. [29] have shown in their research a way to transfer agile methods from software product development to physical product development by systematically introducing alternative technologies to produce physical prototypes. By using a computer-aided design (CAD) and finite element simulations (FEM), it is possible to produce and test virtual prototypes (VP) already in the development phase. This enables a faster development process and is the basis for a rapid review of solutions, their evaluation and, last but not least, the generation of new ideas within the development team itself. Vinodh et al. [30] also state that the use of computer-aided engineering (CAE) and rapid prototyping (RP) tools enables the use of agile methods in the development of physical products. These tools enable rapid development iterations and testing, offering greater flexibility and leading to significant cost reductions. The APD method can also be used in industrial manufacturing. According to the findings of Potdar et al. [10,31], this is a key component in bringing products to market quickly and successfully. Stare [32] finds a systematic lack of implementation of ADP in physical product development (see also Raj et al. [33]). As Chen et al. [34] argue, the implementation of APD can be particularly beneficial for small and medium-sized enterprises (SMEs).

### 1.3. Research and Development Challenges in the e-Mobility Sector

Due to the decline in fossil fuel consumption and increasing pollution, electric drives are becoming the most widely used alternative to regular internal combustion engines. Electrification and the share of electric drives varies in transport systems by region and sector. The lowest share of electric drives is found in agriculture and wood processing. Caban et al. [35] note that the biggest problem with electric tractors is that they only allow a few hours of work, which is only sufficient for orchards or greenhouses. Hybrid systems composed of fuel cells and batteries are mostly used in trucks and buses, where one of the biggest obstacles is the uncertainty of hydrogen supply and the high cost of hydrogen production [36]. Private car electrification is currently dominated by the so-called battery electric vehicles, where batteries are a key factor for the autonomy of the vehicle [37,38,39]. Electric bicycles represent the largest share of the e-mobility market. Many studies point to the importance and benefits of this type of transportation [40,41,42,43,44]. An attempt to combine electric cars and electric bicycles is the so-called E-3 Kolka, which has two wheels in the back and one in the front [45]. E-scooters and e-skateboards are also becoming more popular. Such new mobility solutions are becoming more popular among shared mobility providers and are also a new social trend [46]. In his study, Gogola [47] notes that with the emergence of electric transportation systems, speed is increasing and points to poor infrastructure. Given all these rapidly changing trends, agility is essential in the development of such products.

Apart from the mechanical power source in e-mobility products, i.e., the electric motor, a crucial part of the system are the power transmission components. Here, gears are, in general, the most dominant and widely used components for transmitting rotational motion and power, as well as achieving various required transmission ratios. High-performance gearing design, especially when non-metal materials such as polymers and composites are used, is a complex task that requires extensive know-how and the involvement of numerous digital and numerical tools such as CAD, Finite element analysis, standards-based computational tools, etc. Gears are prone to many different damage modes during operation, induced by applied load conditions, selected geometries, materials, lubrication conditions [48] and even secondary influencing factors such as housing vibrations [49]. Depending on these factors, non-metal gears mainly fail due to the following mechanisms: wear [50,51,52], root fatigue [53,54,55], flank fatigue [56,57], thermal overload [55,58] and pitting [59]. In metal gears, while thermal failure is not a crucial issue, the other failure mechanisms are indeed possible and often observed in addition to scuffing [60], spalling [61] and other. Often, research studies are limited to analyzing single gear pairs with specific material and lubrication condition selections. In real-life applications, however, the powertrain is often composed of multiple stages and complex gear configurations such as, e.g., planetary gearings. Several studies have focused on the whole system analysis of multistage gears or planetary gear transmissions using various experimental and numerical approaches [62,63,64,65,66]. Numerical analysis methods offer an indispensable tool for the implementation of agile product development in powertrain design. Experimental diagnostics methods are also crucial in assessing the developed product in the physical prototype form. However, in order to achieve optimal, i.e., the shortest possible development cycles, it is crucial to perform testing and material characterization on a base component level (following relevant standards and guidelines) before or during the initial development phases, thus obtaining all the required material data for a robust powertrain design, and minimizing the number of required physical prototypes.

This paper presents a real-world project related to the gearbox drive system with polymer gears. The central drive powertrain system is used in an e-bike. Throughout the project, the authors were directly involved in the design process. The project work required a number of changes to product requirements due to customer needs and demanded a high level of adaptability from the development teams involved. The APD method with an integrated Scrum framework was identified as the appropriate approach, even though some adaptations were required for mechanical products. Based on the experience gained in the project, a generally applicable method based on agile product development was prepared. Mechanical and mechatronic systems were the target area of application.

## 2. Project Description and Applied Methods

The research critically reviewed the development and mechanical design process of the e-bike drive system. The market for e-bikes has rapidly evolved in recent years. New models of e-bikes are to be launched in the spring. This results in additional requirements for accelerating development activities. Due to the high expectations, agile product development methods with specific adaptation were used. The use of APD methods was critically evaluated at the end of the project. Corrective measures for better project management were defined based on systematic analyses under various aspects. 

A key concept of agile methods is rapid feedback and testing of products. When developing physical products, this is of course not possible due to financial and time constraints. We tested virtual prototypes and different configurations of mechatronic prototypes to find the optimal combination.

In the following pages, several key methods are presented that together formed a comprehensive APD framework for the entire project at hand:

1. Analysis of the team composition based on the Scrum framework.
2. Review of the APD-based product development process and the influence of sudden specification changes on the process.
3. Specifics of polymer gears development following the APD framework.
4. Critical review of the communication model in correlation with the considered Scrum method.

The way the APD methods were used was critically evaluated at the end of the project. The review also represented the foundation on which the agile development guidelines for mechatronic products, subsequently presented in Sect. 3, were built.

### 2.1. Project Background

The goal of the project was to develop a new e-bike drive system (powertrain). The dynamic market for electric bicycles led to several changes in technical and commercial requirements during the project implementation. Project activities were carried out in a manner characteristic of APD in combination with concurrent engineering. It should be emphasized that at the beginning, a very tight time window was given and the requirements in terms of volume and required power of the powertrain could not yet be met. 

Competitive products were benchmarked, and several products analyzed. Some of the existing solutions used toothed belts, others gearing. Gears made of plastic have several advantages and were therefore considered a real option from the start. Plastic gears run quietly, require no lubrication and can be manufactured at a competitive cost. However, plastic gears can only be loaded to a limited extent and are sensitive to elevated temperatures. Therefore, the project required specific skills to develop polymer gears and test their durability.

### 2.2. Team Composition

**Figure 1:**
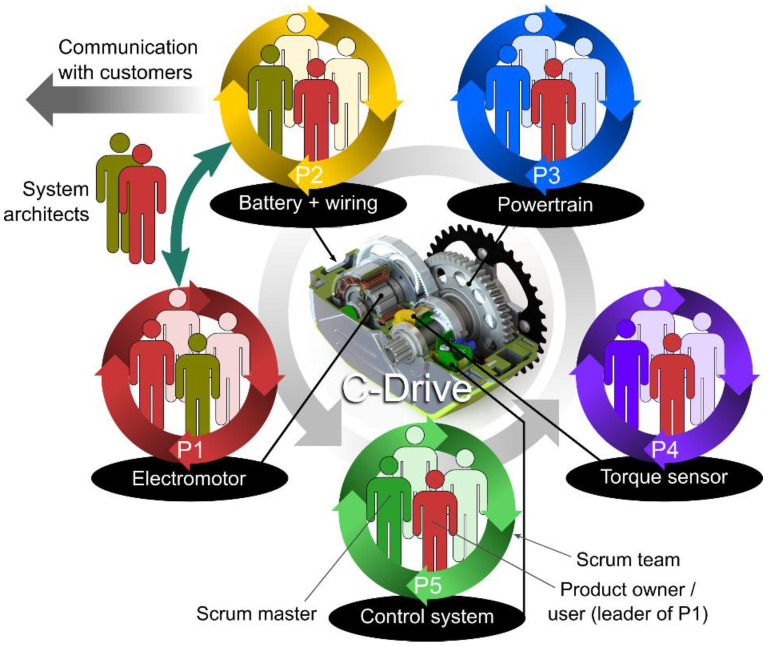
Composition of the sub-teams for various powertrain modules.

Sub-teams were formed for each module of the powertrain, as shown in Figure 1, early in the project. The sub-teams were essentially Scrum Teams (STs). Since the modules are not independent of each other, regular communication between sub-teams was practiced. 

The tasks of the project sub-teams (i.e., STs) were as follows:

- Sub-team P1: Project coordination, system design, motor design with electronic commutation, powertrain durability testing.
- Sub-team P2/customer: system design, power source and wiring, analysis of customer requirements (bikes manufacturers)
- Sub-team P3: Powertrain R&D (including polymer gearbox)—the authors were part of this team.
- Sub-team P4: Development of the torque sensor
- Sub-team P5: Development of electronics and control system

This paper authors were active in sub-team P3 that was working on powertrain, and the presented study relates to the development of a mechanical assembly with polymer gears. 

### 2.3. Modifications to the Technical Requirements

Sub-team P2 created the original technical requirements for the powertrain. The first modification to the requirements occurred only five weeks after the original specification was established. After the virtual prototype was presented to the powertrain customer, several new requirements surfaced. The virtual prototype included product architecture, mechanical components and dimensions. The presentation of the prototype allowed for a better understanding of product structure, functionality and competences of the powertrain team. The technical requirements became much more demanding. The required product durability was extended by 10%, while the permissible dimensions and weight of the drive have been reduced—length by 12%, width by 7%, height by 4% and the total weight by 25%. 

Seventeen weeks after the first modification to requirements, a second change followed, increasing peak power by 100%. At the same time, the permissible housing temperature has been reduced by 25%.

Generally, the technical requirements upgrade occurred at the end of the sprint, after the presentation of product development was performed. The second modification was triggered by a benchmarking analysis. One of the products on the market offered a powertrain with better performance than in the original technical requirements.

When the technical requirements changed, the traditional product development process would be shifted back to the beginning. Using agile methodology allowed the Scrum team simply to start a new sprint. Product development activities in different sub-teams were affected by mutual dependency between sub-modules. The integration of a torque sensor, connector sub-assembly and electronics at different stages of development led to additional changes in the powertrain assembly. 

### 2.4. The Development Process

**Figure 2:**
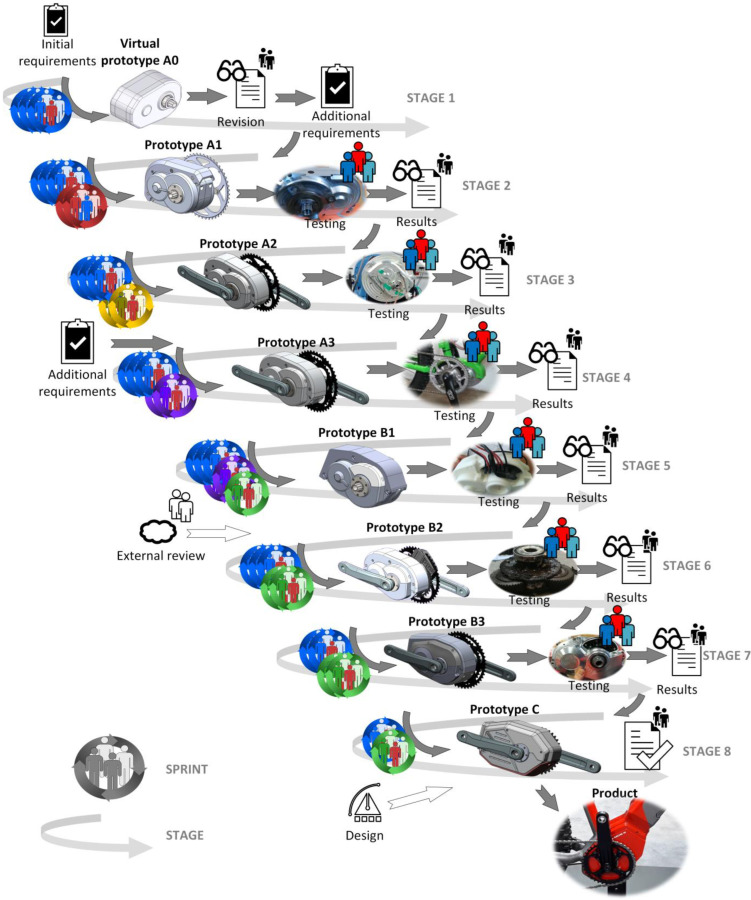
The drive system’s development process.

The rough representation of the activities in the product development process is shown below (Figure 2). After each phase, a virtual (after the first phase) or mechanical prototype was created. Several sprints were conducted in each phase.

After confirming the initial customer requirements, a conceptual design of the powertrain, designated VP A0, was created. During the development process, several numerical simulations (FEM analyzes) were performed, and appropriate standards were applied in the calculation of the components. Then other components were included (couplings, sensors, housings, etc.). 

**Figure 3:**
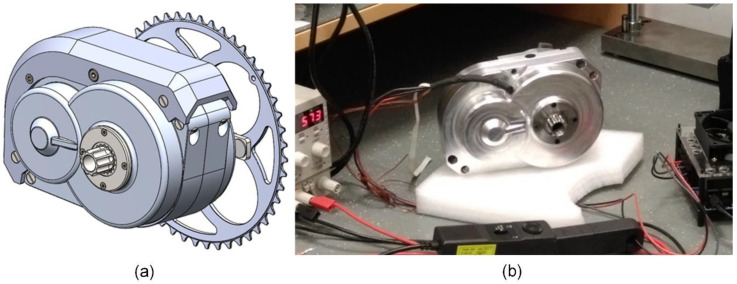
Prototype A1: (**a**) virtual prototype—CAD model; (**b**) functional testing of the A1 prototype with external electronics under no external load.

After the modification to requirements were specified, the sub-team P3 for the powertrain started the new sprint by implementing the required changes. After numerous improvement steps, the powertrain design was ready for the first working mechanical sample—prototype A1 (Figure 3). The powertrain sample was used for functional and durability testing. During the test, various operating parameters were measured, such as the temperature at different points and sound spectrums. Manufacturing technologies were adapted to the number of parts produced. The drive housing, the part with the most complex geometry was produced on a 5-axis CNC milling machine. The materials used for gears in prototypes were always the same as they were supposed to be in the final version of the drive for serial production. A realistic precondition on noise level, mechanical properties and temperature measurements is very important. Polymer ring gears were manufactured with injection molding already in the prototyping phase. Planetary gears were manufactured by injection molding (basic geometry) and after that, by cutting with the hobbing tool for better accuracy [67].

**Figure 4:**
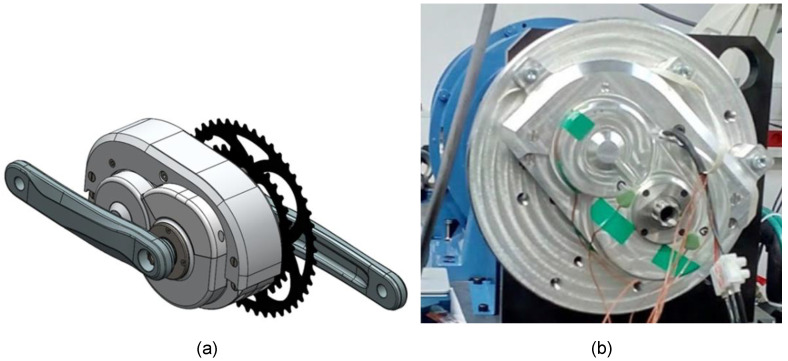
Prototype A2: (**a**) virtual prototype—CAD model; (**b**) testing of a prototype under load at a test site with a brake.

A thorough analysis of the successfully completed prototype A1 led to the identification of critical deficiencies that needed to be addressed in subsequent iterations. These concerned both the assembly and functionality of the current solution itself. At the same time, the design had to be adapted to allow mounting of the array connectors and electronics required to power and control the drive, as well as the torque sensor. While the following prototype iteration A2 did not yet incorporate the above components, the housing was already modified to allow these components to be inserted, as shown in Figure 4, and connect the drive to a customized bicycle frame.

The A2 prototype met all the set requirements, however, at the time of introduction, a competitor just re-leased a new product on the market, which noticeably surpassed the drive performance set in the current specifications of the presented project. This led to a second change in specifications, which now included 100% increase in peak power and a lower overall weight of the drive, leading to significant problems at that stage of the project. 

**Figure 5:**
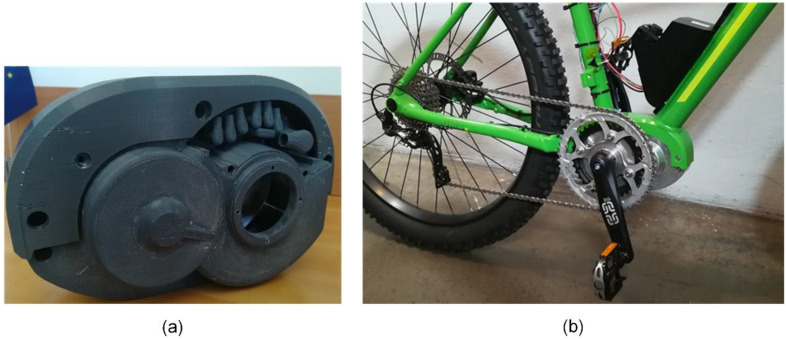
Prototype A3: (**a**) 3D printed prototype to test the suitability of a connector set; (**b**) functional prototype tested on a bicycle.

Since the electric motor did not provide sufficient peak power at the present gear ratio, it had to be modified by introducing stronger magnets, and the gearbox had to be changed to increase the gear ratio while maintaining the overall volume and further reducing the mass. This task was naturally handed over to the ST responsible for the mechanical assembly (i.e., ST 3), which managed to increase the gear ratio by 25% while maintaining the same volume. The two-stage gearbox now included a pair of steel gears on the second stage, breaking away from the plastic version previously used for the second stage driven gear. While the use of steel could bring with it an increase in the mass of the drive, this was offset by a reduction in the face width of this gear and various other design changes that were now possible thanks to the newly gained space previously occupied by the larger plastic gear. These changes, along with a newly developed custom torque sensor and power and control electronics, were introduced in physical form in the prototype A3 (Figure 5). The latter was used to test the mechanics and control system at the newly set power levels. Heat loss-related temperature-rise and noise level measurements were also performed here.

**Figure 6:**
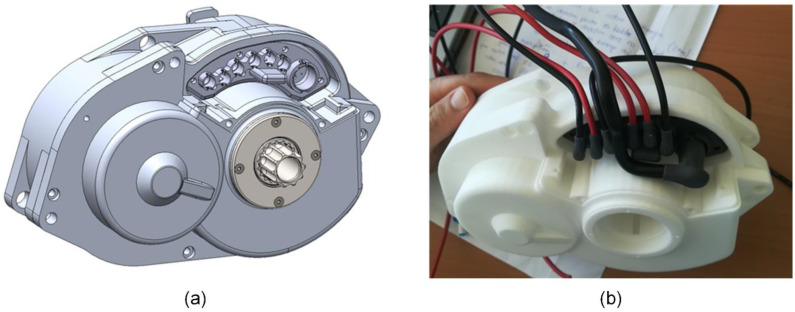
Prototype B1: (**a**) virtual prototype; (**b**) a 3D printed prototype B1 to test space for connectors.

After confirming the updates introduced in A3 and a modified mounting system requested by the customer, the B phase of prototyping began with prototype iteration B1 (Figure 6). Some challenges related to transmission reliability led to an external review of the solution by a selected R&D partner. Working with the auditor, several important corrective actions were taken (introduced in prototype B2) that significantly improved the overall drive system in terms of durability, peak power transmission, and smoothness, and enabled the new functional requirements to be met. Finally, the external industrial design of the drive was added. The first design iteration was introduced with the B3 prototype, which was presented to e-bike vendors. The customer noted their feedback and requested the implementation of an updated design, which was created by an external industrial design partner. The result was the C prototype, which was eventually approved for mass production and installation in the selected e-bike brand.

#### Development of Polymer Gear Transmissions 

Polymer gears were indispensable due to technical requirements for low noise levels and low weight, and competitive costs. Polymer gears enable reliable operation even without lubricant. There is not only one argument for selecting polymer gears, but a sum of advantages in comparison to disadvantages and other technical options. However, vibration damping and reduced noise levels are the main reasons for selecting polymer gears. Simultaneously with the development of the entire drive, intensive research and development took place in the field of polymer gears. Polymer gears have a limited load capacity. The first stress level showed a gap between the required load and the mechanical properties of the polymers. There was a large number of potentially interesting polymer gear materials. However, several material properties such as fatigue strength, wear and coefficient of friction, which are important for the design calculations of polymer gears, were not available in the material data sheets. Therefore, gear tests for different material combinations were carried out before and during the development of an optimal gear drive. Plastic gears for testing were manufactured by injection molding. Durability tests have not shown any significant difference between cut and injected molded polymer gears. The steel gears for testing were manufactured by cutting [68]. Gear testing is very time-consuming, especially when several different material combinations are tested under different test conditions. By using the accelerated testing method, the time and cost of gear testing could be significantly reduced [69]. Poorer mechanical properties of polymer gears at higher temperatures are an important limitation [67,70]. Advanced numerical simulations and experiments have been performed to better understand heat generation and dissipation [71].

**Figure 7:**
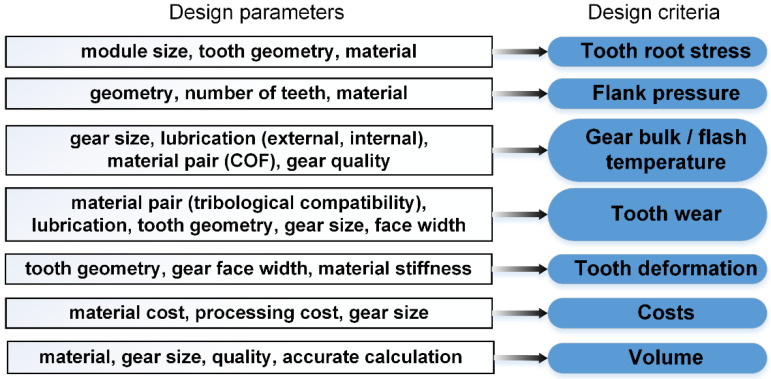
Gear pair design parameters and how to influence design criteria.

With polymer gears, there are several limitations due to the material properties that need to be checked during the design process: root and flank strength, temperature, wear, deformation, gear quality and final installation quality (Figure 7). All of the above criteria were considered in the design of polymer gears. The most critical criteria and failure mode are determined by the level of load, the pair of materials used and the operating conditions (rotational speed, ambient temperature, lubrication). The design of polymer gears is a complex iterative procedure considering and balancing several criteria [48]. The optimal design and calculation of different variants have been supported with a multi-criteria function [48]. The multi-criteria approach considers the optimal balance between all mentioned criteria depending on the operational conditions and design constraints.

**Figure 8:**
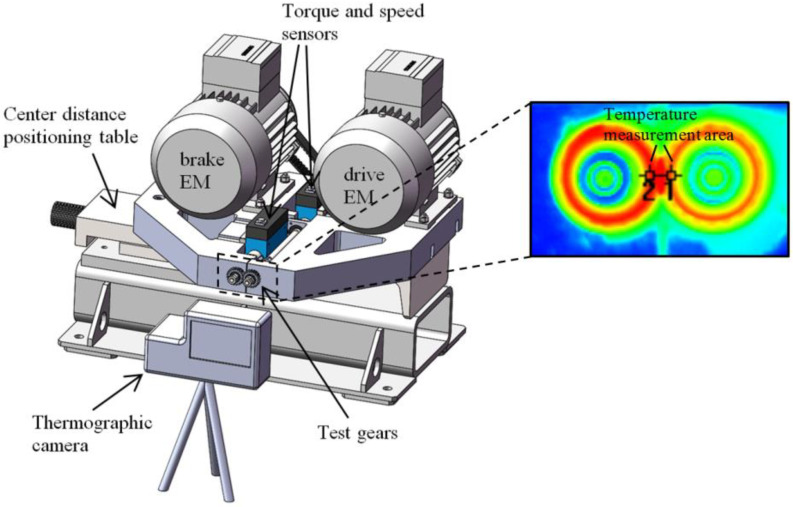
Durability gear testing on test rig with control of speed, torque, and temperature.

The product development team already had expertise in the field of polymer gears. Additional research was conducted on standard test rigs and standard gear samples. Polymer gear design guidelines and material properties were used as input for the design of the drive. Parallel to the prototyping, durability tests were carried out on a test bench (Figure 8). The aim was to determine material properties for the selected pair of materials, which are important for calculating the durability of the gears.

**Figure 9:**
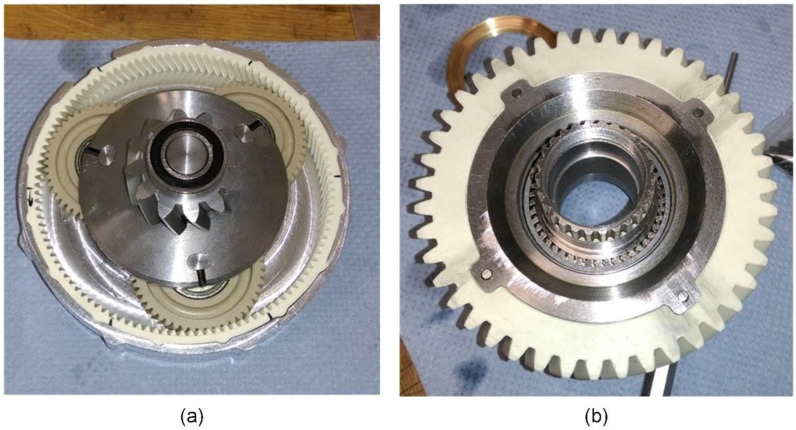
Polymer gears: (**a**) 1st stage—planetary gear; (**b**) 2nd stage—driven gear.

The pinion is connected to the shaft of the electric motor and is made of steel. The pinion meshes with three planet wheels made of plastic (Figure 9a). The planet gears rotate at a high speed, so it is important to dampen the vibrations with plastic. The ring gear is also made of plastic. The gear pair on the second stage initially consisted of a steel pinion and a plastic gear (Figure 9b). Due to the changed requirements (maximum load) and the limited volume, the material of gear (Figure 9b) was later changed to steel.

**Figure 10:**
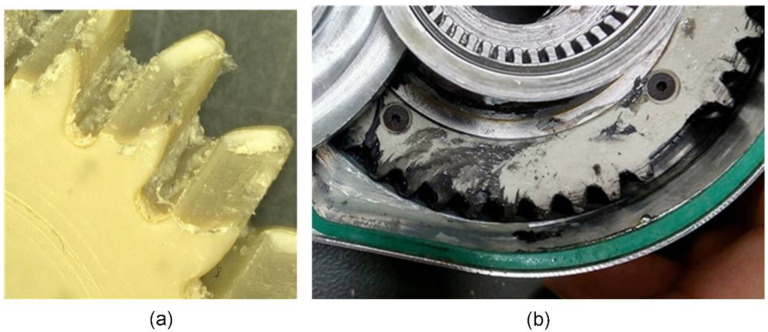
Failure mode of polymer gears: (**a**) wear of PEEK gear paired with steel gear; (**b**) tooth fracture of gear 2 (stage 2).

Wear of polymer gears (Figure 10) was identified as the dominant failure mode. This was the trigger for additional research activities to reduce wear and extend the service life of polymer gears. The additional treatment of metal gears with trovalization significantly reduces the wear of polymer gears and extends the service life [68].

With the correct design of the gears and accurate data of the polymer material, we were able to significantly shorten the development cycle, reduce the number of iterations of the physical prototypes and thus increase competitiveness in product development. Various measures have been introduced to extend the service life. Prototyping entire drives is even more expensive and time-consuming. It is important to reduce the number of iterations as much as possible. By calculating polymer gears and reliable material data, the number of prototypes required could be significantly reduced.

### 2.5. Communication

During the project, several areas of communication were important: communication between team members, communication between individual teams and communication with the drive’s customer. The customer also communicated with bike suppliers and battery developers, as well as with end distributors and resellers of the electric bikes, who knew best what the customer wanted. The information was passed on to the development teams of the individual drive sets in the form of modifications.

Inter-partner communication is critical, especially for teams working on the same product line that are not in the same location. The members of the development teams for the electric motor, electronics, connectors and torque sensor were in the same place. In contrast, the members of the team developing the mechanical transmission were in three different locations.

The members of the mechanical transmission development team had daily online communication. At least once a month, the team members also met on site. At a critical stage in the development of the version B prototype, the members of the mechanical transmission development team moved to the same location for two months. This was a critical part of the project where problems with the prototypes arose and at the same time, the final details for the production of the later versions of the prototypes and also for series production had to be determined. In this phase, on-site communication played a crucial role in speeding up the change and solving problems.

Due to the geographical distance, communication with the other partners took place mainly online, with each of them meeting physically at least once during the project. During the development, regular communication with the drive’s client took place, mainly online due to the distance, but on-site meetings were also scheduled several times. During key phases of the project, improvements to the virtual prototypes were presented to the client in weekly video conferences. Before the meeting, the virtual prototypes were evaluated and tested by the team members. After each meeting, the necessary changes and additions were determined.

Document sharing within a company is usually governed by a PLM system. In development teams composed of members from different companies, the members usually did not have access to the PLM system of all companies, but only to the one in which they are employed. In the development task studied, the exchange of documents took place via a server. Which information and documents the individual members had access to was regulated by access rights. The latest version of the documents always had to be uploaded to the server. This is important so that all members are aware of all the information and so that changes to the design and to individual documents are traceable.

Access to the latest revision of documents was recognized as an open issue that could be improved. A common server was used for storing 3D models and documents. However, it was important to update the files on a regular basis and it was a human factor. Each document had the basic meta-data such as author, date of creation, date of uploading, revision. It helped to assure traceability between different versions of documents. There were several security measures to assure controlled access to the documents: (1) Each of the team members signed an NDA document. (2) Research activities were running in dedicated rooms; random visitors were not able to access documents. (3) Access to the server was controlled with username and password. The document management software enabled access traceability to documents. (4) Regular backups and storing of backups at safe locations. Some more advanced measures such as encrypted files and encrypted communication were not applied. The awareness of team members was the main pillar of data security.

## 3. Results

Following the completion of the central drive development project, the process was critically reviewed in terms of operational efficiency, communication, information gathering, and design testing. The review was conducted within the available agile methodology and Scrum framework guidelines. Several issues were identified with the project execution, but also with the available guidelines, as most of them were only partially applicable to the type of project described. Using the obtained review insights, new agile development guidelines, tailored to mechatronic product development were developed.

### 3.1. Identification of Key Problems in the Development Process

The key deficiencies that affected the overall performance of the development process are:

1. Testing of the developed product using prototypes is of paramount importance. Producing a physical prototype is time-consuming and costly and can result in project delays if a large number of prototype iterations are required. Virtual prototypes can often successfully replace physical prototypes, provided that the underlying assumptions of the used physical models have been suitably validated.
2. While initial specifications were accurately defined, these often changed based on new insights gained by the customer either following their internal market analyses or after the presentation of intermediate physical prototypes by the development team. Several changes occurred fairly late in the project resulting in further delay. Using virtual prototypes to convey new important design changes can help the customer understand more quickly what their actual requirements are and iterate to the final solution faster.
3. To confirm that newly introduced solutions are technically suitable, virtual prototypes have their limitations, and physical testing is often required. Partial physical prototypes proved critical to determine if a function (e.g., one-way clutch transmission, planetary gear, sealing, etc.) was successfully met. To test and confirm critical components and sub-assemblies more quickly with partial physical prototypes, access to rapid prototyping tools that enable component production of comparable quality to conventional production methods is essential.
4. The response to changes in customer requirements was often too slow. Adapting to intermediate specification changes can only be conducted in a quick and responsive fashion if modern CAD/CAE software tools are systematically used to develop and numerically test new VP iterations and then transition to physical prototypes. Using suitable parametrization methods for quick model updates and numerical simulation checks, combined the model-based definition approach (MBD) to transition seamlessly from model to production can yield quicker prototype iterations.
5. Only after the mechanical system of the e-bike drive was developed, the electronics necessary for system power supply and control could begin to be tested. This could have been avoided, i.e., the electronics could be tested in parallel with the mechanics, if a suitable prototype testbed replicating the e-bike operation conditions was available for this purpose.
6. While regular meetings were scheduled during the project to help keep all members of the various STs informed, they were often too broad in scope, regularly including all or most of the STs involved and covering a wide range of topics that often did not directly affect all STs. Broader meetings should be time-limited and only serve to define what needs to be done, not discuss problems. Issues are then discussed in more detail within each ST or among the STs involved.
7. The project backlog and reviews of each development cycle—both in terms of design updates and new knowledge gained—were often poorly managed, contributing to slower than optimal project progress. Implementing appropriate (web-based) tools and methods for regularly updating and sharing the latest relevant information is especially important when the parties involved are from different organizations across wide geographical areas. In the project described, the flow of information proved to be suboptimal, as information was not exchanged directly and quickly enough between the parties involved.

### 3.2. Agile Approach to the Development of Polymer Power Transmissions

The issues just described were identified only after a thorough analysis of the project, most of which was conducted after project completion. In conjunction with a comprehensive literature review, several new rules and guidelines—based predominantly on agile development principles—were distilled from all the information gathered. These guidelines, described below, could help make the physical product design process more efficient and lead to superior products.

1. The product is divided into individual sets that are interdependent and ultimately make up the entire system but can also be developed individually and simultaneously to a certain extent.
2. Teams are composed of members with specific skills required to develop each set. Work on individual sets should take place within an appropriate agile framework. In the case studied, Scrum proves to be a suitable basis.
3. The product development team needs to be supported with key skills such as polymer gear design. Necessary research must be conducted in parallel with a simplified and generic model to reduce time and complexity (if possible).
4. The results of each iteration/sprint must be evaluated accordingly. Numerical simulations (Finite Element Analysis—FEA) and engineering tools such as FMEA (Failure Mode and Effects Analysis) and DoE (Design of Experiments) can be employed to assess the virtual prototypes. Features that cannot be evaluated on the virtual prototype are tested on a physical prototype. The physical prototype needs to be adapted to the testing purpose.
5. Continuous product testing is essential. This is divided into the following two categories:

A) Functionality testing

 ▪ Functional testing is carried out at four levels and takes place before testing at the customer’s premises: (I). Virtual prototype testing, (II). Testing of partial physical prototypes (e.g.,: Testing polymer gearboxes), (III). Testing full physical prototypes of each stage and (IV). Testing of the whole product (full physical prototype—combined results of all stages).
 ▪ A full physical prototype is required for functional testing of the product. The prototype merges the results of successful phases of all Scrum Teams. The production of the prototype is time consuming and usually involves high costs. Individual product subassemblies are interconnected but are not always needed for testing in all phases. Some tests can be performed on prototypes that only cover the R&D work of a single scrum team or a limited number of Scrum Teams.

B) Product acceptance testing with potential customers

 ▪ The result of each sprint is an updated virtual prototype. A set of fully functional virtual prototypes can be assembled into a comprehensive virtual prototype that can be shown to the customers (augmented or virtual reality can be used at this stage). By reviewing the customer feedback, the necessary changes that lead to a higher product value are identified. Customer feedback must be taken into account in the next sprint. It is especially important to communicate with customers in the earlier phases and present updated results (and thus determine what customers want).
 ▪ Identify modules or submodules that can be further developed while waiting for production and test results of a physical prototype. Optimal parallelization of development tasks is the goal.
 ▪ There are several critical issues to consider when developing a completely new, complex physical product. If ST does not master these, it may not be able to meet the required product specifications. External revisions can provide new solutions and help bridge the critical points.
 ▪ Information must be communicated comprehensively and as quickly as possible, without unnecessary noise. Short chains of communication usually prove to be the best.

**Figure 11:**
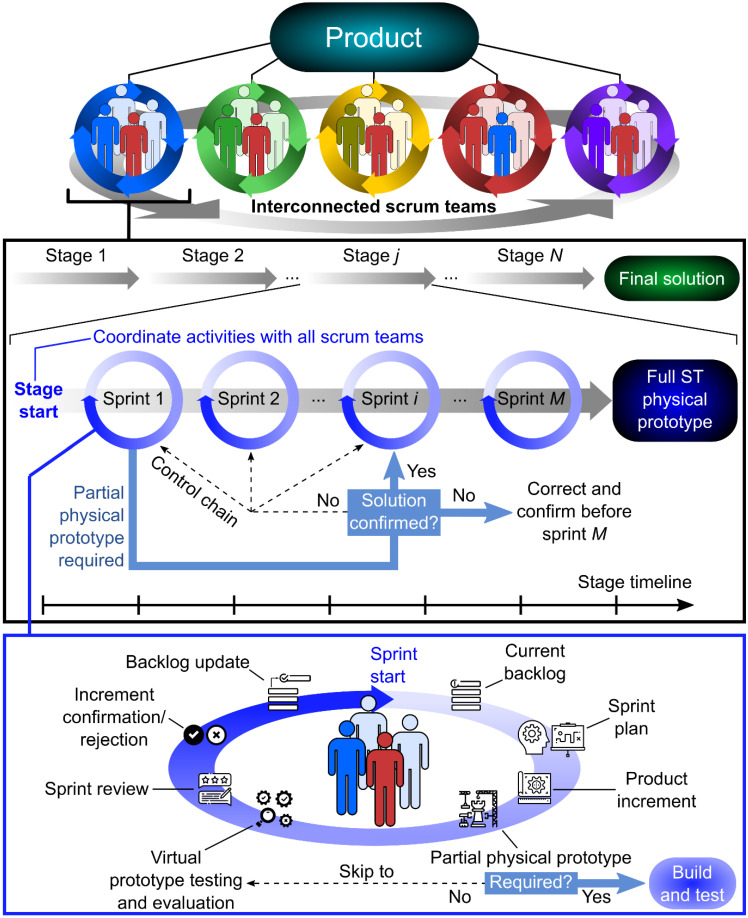
Scrum team workflow based on the Agile product development process.

Accordingly, we have developed an updated model of agile development of mechatronic products using the Scrum framework (Figure 11). Product development is divided into specific areas and corresponding Scrum teams are formed, which are interconnected and at the same time autonomous in their area. The development is divided into individual stages. Within each stage, the required number of sprint cycles is carried out. The result of each Stage (ST) is a functional ST prototype. 

**Figure 12:**
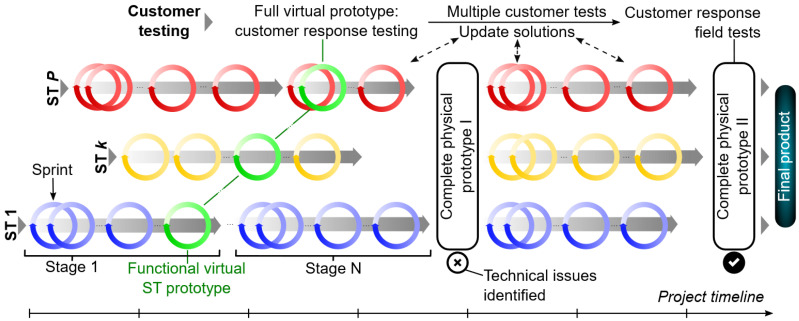
Exemplary project timeline, based on the Agile product development process.

Each sprint requires the internal structure as shown in Figure 11 (lower part of the figure). The sprint starts with the sprint plan and continues with product development. The decision whether to create a physical prototype or not is made in the next step. The VP and/or the physical prototype are tested and evaluated. The result of the sprint review is an input for the next sprint. It is important to record all important information in the backlog. An example of a timeline can be found in Figure 12.

### 3.3. Agile Approach to the Development of Polymer Gears

**Figure 13:**
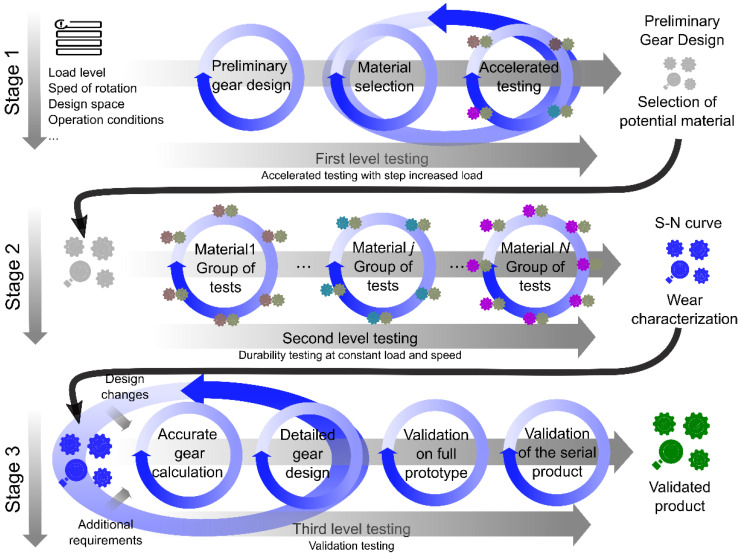
Accelerated polymer gear design procedure integrated in the Agile product development process.

Taking into account the observations made during the project, the previous experience of the development group in the field of polymer gears and the principles of agility, we propose the model of polymer gearbox development shown in Figure 13. The proposed accelerated method for polymer gears has several test stages resulting in a reduced number of tests and providing reliable material properties for the calculation of gears for various applications. By conducting accelerated step tests and lifetime tests, material data required for gear design calculations were generated. The key material characteristics were: (i) Material’s fatigue strength in relation to the temperature and number of load cycles. (ii) Coefficient of friction for the selected specific material pair. (iii) Wear factor for the specific material pair. An endurance test rig for polymer gears is required to perform accelerated gear tests. The procedure consists of several steps:

1. Preliminary design of the gear based on the input data (simple calculation of the gears, material selection). If the selected polymer material pair already has known material properties, the procedure can be continued with Stage 3.
2. Accelerated (step) tests. Preliminary step tests (increasing the torque level in several steps). The step test is a fast prediction of tribological properties and polymer strength and helps to identify the most promising material pairs, which are then followed up in life tests (durability tests). Coefficient of friction can be determined from operating parameters and measured temperature as presented in [69].
3. Lifetime tests on a test bench with standard size gears (at selected speed and torque). The test is carried out with the pair of materials and under conditions close to those of the final product (speed, temperature). Wear, coefficient of friction and durability are properties, which depend on selected pair of materials. Durability tests of gears are the best indicators of material properties, which are important for the calculation of gears [72]. Material’s fatigue strength can be determined from the tested load level and gear geometry. The number of load cycles can be calculated from the test duration before failure and speed of rotation. Wear factor is assessed from measuring wear during durability test as presented by Zorko [68].
4. Detailed calculation and design of polymer gears: well-defined material properties (Stage 2) enable reliable calculation of gears. This reduces the number of prototypes required.
5. Testing of gears in the final application (product validation). The production of tools for injection molding is expensive and time-consuming. It is important to perform all the previous steps well and create the tools only once.

When developing polymer power transmissions, there are several performance enhancements possible. The first option is gear shape optimization. Most mass-produced polymer gears are injection molded, hence, the manufacturing cost is not dependent on the gear profile shape. Therefore, profile modifications such as enlarged root rounding or fully rounded roots can be easily applied. Furthermore, special non involute gear geometries with increased tooth bending strength and reduced specific sliding can be used, without the need of special cutting tools and additional costs [73]. The second possibility to enhance performance is to use better materials. Gear designers can go up the polymer pyramid to high performance engineering materials from the PEEK family. Such a performance enhancement is usually also accompanied with a substantial cost increase. Then, materials can also be improved by different fillers such as fibers or internal lubricants, and laminated composites have also shown great potential [74,75,76]. The third possibility is a combination of the previous two and of course there are also other possibilities related to material processing when producing the gears [77].

## 4. Discussion

The conclusions of this research are consistent with the experiences of several other authors who have attempted to integrate agile methods into the physical product development process, such as Cooper and Sommer [17]. The physical product development process has limited flexibility and is closer to the stage-gate process structure in many requirements. Agile methods from software projects cannot be easily copied and applied. The relatively high unpredictability of the market, customers and technology requires flexibility in the physical product development process. The application of selected agile tools and methods is therefore necessary and very helpful.

Salvato and Laplume argue that the agile approach has a negative impact on the efficiency of project resources [20]. This is true for predictable product development projects at the variation design level, where team members can work on several projects at the same time with high productivity. In our case, we had a new and complex product, and it was necessary to dedicate the core of team members to this project only. The level of unpredictability was high, so the use of agile methods (Scrum, Sprint, Backlog) was an important advantage for the project. Our project experience is in line with the recommendation of Baschin et al. that the choice of agile method should be tailored to the constraints of the organization [25]. We have adapted the agile tools and methods as well as project management to the needs of the project.

Hendler and Boer argue that binding decisions must be made early in the physical product development process [21]. The authors of this article agree with this assertion. It would be very helpful to define the technical requirements for the drive early in the project. This would speed up the development process and make the activities more predictable. However, real-life circumstances are often very different. Without a change in product specification, the new drive would not meet key customer expectations and market needs. Therefore, it is important to allow flexibility during the project.

If we consider the presented development process in the context of the presented guideline, some remarks can be formulated. The product was divided into several interdependent sets, which could be developed individually to a certain extent. The Scrum Teams were in continual communication with the customer, who was incidentally a member of the development team one level higher. The entire development process was guided from the beginning by the wishes of the end customers. These were identified by project managers and system architects and passed on to the STs.

How strong prototyping needs to be in physical product development is an important question. Zink et al. argue that prototyping is the key aspect of the agile approach and have established criteria for the use of physical prototypes [28]. Reichwein et al. suggest implementing additive manufacturing technologies in the development process and combining them with virtual prototypes [29]. The powertrain team was highly competent in numerical simulation. Virtual prototypes were helpful and were used frequently during the project. However, testing of the physical prototypes ended with several unexpected failure modes and contributed significantly to a better understanding of how the powertrain works. Therefore, several physical prototypes are essential when developing a new generation of products. Virtual prototypes are sufficient if we can first validate them with physical prototypes, e.g., for the variation level of the design. The 3D-printed prototype was used to test the space for the connectors, but not for other functional properties. In general, the prototyping used a manufacturing technology that ensures similar mechanical properties as the later series’ production. The functional and full load tests were necessary to identify weak points in the design.

The STs consisted of a fixed number of members, but experts were brought in depending on the phase and project needs. The need for specific knowledge and a larger number of staff was only present in individual development phases. Individual members of ST could contribute in the phases where their specific knowledge could contribute to a better product. The results of each sprint were evaluated by all Scrum members and presented to the customer who was most familiar with the e-bike user’s needs. The set of activities foreseen in sprint planning also defined the duration of each sprint.

Sprint planning was conducted by the members of ST before the next sprint was started. The aim was to identify the requirements to be solved in this sprint. At the end of the sprint, the results were reviewed and presented to the client. Together with the customer, the design review was conducted and the requirements for the next sprint were defined. After successive sprints, the result was verified on a prototype. The prototype could be partial models—pure design or partial functional prototypes. The individual product assemblies are interdependent, but it is not essential to test them all in each phase. For example, version A prototypes were used to test mechanical performance and had an external power supply and control electronics, while the torque sensor and connector assembly were not included at this stage.

Different projects and products require specific competences. In the case of the drive, the external partners for the control system and the torque sensor were included in the project team. The key competence of the scrum team for the powertrain was the knowledge of polymer gears. It was crucial to split the whole project into tasks that could run in parallel. The results of the parallel proficiency tests on the test bench were used for the polymer gearbox calculations and the powertrain design. This reduced the number of prototyping iterations needed, contributed to an optimized design and shortened the overall development time of the drive.

### Limitations

The project carried out has specific characteristics, such as the degree of complexity, the experience of the partners involved and the competences of the members of the project team. The authors have tried to set guidelines for the agile approach in general. However, each company and each product has specific requirements, and each team has specific competences, so agile tools and methods have to be applied individually. This is in line with [25]. The mechanics team developed a drive for the first time. The results and guidelines would probably have been different if the team had already completed several similar projects. The unpredictability of the requirements was high and troublesome for team members. Therefore, the application of the flexible agile methodology was appropriate. The optimal set of agile tools and methods and the way they are implemented need to be customized for each organization.

## 5. Conclusions

This study was conducted during an industrial development project. The development was based on a combination of Scrum-based APD and concurrent development methods, which enabled immediate address of all challenges, resulting in an excellent final product and a satisfied client and end customer. Regular communication with the client and frequent iterations/sprints related to presentations of the results proved to be crucial for early introduction of changes.

Physical prototypes are essential for testing and validating the functionality of mechatronic products. Compared to software development, physical products usually require the creation of several physical prototypes in addition to virtual prototypes. However, this requires a certain amount of time and money. With the right approach, ST can reduce the number of physical prototypes accordingly, while developing certain parts of the product with the production of the physical prototype. When mass producing physical products, it is important that the entire production is well prepared and planned before the final prototype is available.

Prototyping powertrains is time-consuming and costly. It is not acceptable to carry out tests on polymer gears, such as selecting the polymer material or optimizing gear geometry for the entire drive. Systematic polymer tests were carried out before and partly in parallel with the development of the drive. The design of sophisticated drives requires very precise input data on the polymer gear material combinations and durability, which were determined on the test bench for standard polymer gear sizes. The drive prototype is used to fine-tune the design parameters and for product validation. It is important to identify key technologies and competencies and integrate them into the product development team in a timely manner. 

Based on a critical review of the successfully completed project to develop the central electric drive of the bicycle, the study summarizes the most important guidelines for the agile development of mechatronic products.

The authors plan to further use the agile methodology on new product development projects. New development projects of products of varying complexity will contribute to new experiences with the application of agile methods. The goal is to establish criteria and more detailed guidelines for which agile tools and methods should be used in the development of physical products.

## References

[1] Duhovnik J., Tavčar J., Stjepandić J., Wognum N., Verhagen W.J.C. (2015). Concurrent Engineering in Machinery. Concurrent Engineering in the 21st Century: Foundations, Developments and Challenges.

[2] Stjepandić J., Wognum N., Verhagen W.J.C. (2015). Concurrent Engineering in the 21st Century.

[3] Tavčar J., Demšar I., Duhovnik J. (2018). Engineering Change Management Maturity Assessment Model with Lean Criteria for Automotive Supply Chain. J. Eng. Des..

[4] Islam G., Storer T. (2020). A Case Study of Agile Software Development for Safety-Critical Systems Projects. Reliab. Eng. Syst. Saf..

[5] Tam C., da Costa Moura E.J., Oliveira T., Varajão J. (2020). The Factors Influencing the Success of On-Going Agile Software Development Projects. Int. J. Proj. Manag..

[6] Younas M., Jawawi D.N.A., Ghani I., Fries T., Kazmi R. (2018). Agile Development in the Cloud Computing Envi-Ronment: A Systematic Review. Inf. Softw. Technol..

[7] Beck K.M., Beedle M., Bennekum A.V., Cockburn A., Cunningham W., Fowler M., Grenning J., Highsmith J., Hunt A., Jeffries R. Manifesto for Agile Software Development. https://agilemanifesto.org/.

[8] Leite M., Braz V. (2016). Agile Manufacturing Practices for New Product Development: Industrial Case Studies. J. Manuf. Technol. Manag..

[9] Leite M., Baptista A.J., Ribeiro A.M.R. (2016). A road map for implementing lean and agile techniques in SMEs product development teams. IJPD.

[10] Potdar P.K., Routroy S., Behera A. (2017). Agile Manufacturing: A Systematic Review of Literature and Implications for Future Research. Benchmarking Int. J..

[11] Schwaber K., Sutherland J. (2013). The Scrum Guide. The Definitive Guide to Scrum: The Rules of the Game.

[12] Ovesen N. (2012). The Challenges of Becoming Agile: Implementing and Conducting Scrum in Integrated Product Development. Ph.D. Thesis.

[13] Riesener M., Rebentisch E., Dölle C., Schloesser S., Kuhn M., Radermacher J., Schuh G. (2019). A Model for Dependency-Oriented Prototyping in the Agile Development of Complex Technical Systems. Procedia CIRP.

[14] Sommer A.F., Hedegaard C., Dukovska-Popovska I., Steger-Jensen K. (2015). Improved Product Development Performance through Agile/Stage-Gate Hybrids: The Next-Generation Stage-Gate Process?. Res. Technol. Manag..

[15] Cooper R.G. (2016). Agile–Stage-Gate Hybrids. Res. Technol. Manag..

[16] Karlstrom D., Runeson P. (2005). Combining Agile Methods with Stage-Gate Project Management. IEEE Software.

[17] Cooper R.G., Sommer A.F. (2018). Agile-Stage-Gate for Manufacturers. Res. Technol. Manag..

[18] Asmar L., Rabe M., Low C.Y., Yee J., Kühn A., Dumitrescu R. (2018). Framework for the Agile Development of Innovative Product Service-Systems for Existing Physical Rehabilitation Systems. Procedia Manuf..

[19] Gary C. (2004). Agile Project Management: How to Succeed in the Face of Changing Project Requirements.

[20] Salvato J.J., Laplume A.O. (2020). Agile Stage-Gate Management (ASGM) for Physical Products. R D Manag..

[21] Hendler S., Boer H. (2019). Digital-Physical Product Developments: A Review and Research Agenda. Int. J. Technol. Manag..

[22] Hendler S. (2019). Digital-Physical Product Development: A Qualitative Analysis. Eur. J. Innov. Manag..

[23] Smith P.G. (2007). Flexible Product Development: Building Agility for Changing Markets.

[24] Schuh G., Dölle C., Kantelberg J., Menges A. (2018). Identification of Agile Mechanisms of Action as Basis for Agile Product Development. Procedia CIRP.

[25] Baschin J., Schneider D., Huth T., Vietor T. Project-Oriented Selection of Agile Methods for the Design of Physical Products. Proceedings of the 2021 IEEE International Conference on Industrial Engineering and Engineering Management (IEEE).

[26] Schmidt T.S., Paetzold K. Maturity Assessment of Teams Developing Physical Products in An Agile Manner. Proceedings of the 23rd International Conference on Engineering, Technology and Innovation (ICE/ITMC).

[27] Žužek T., Kušar J., Rihar L., Berlec T. (2020). Agile-Concurrent Hybrid: A Framework for Concurrent Product de-Velopment Using Scrum. Concurr. Eng. Res. Appl..

[28] Zink L., Bohmer A.I., Hostetter R., Lindemann U., Knoll A. The Use of Prototypes Within Agile Product Development Explorative Case Study of a Makeathon. Proceedings of the 23rd International Conference on Engineering, Technology and Innovation (ICE/ITMC).

[29] Reichwein J., Vogel S., Schork S., Kirchner E. (2020). On the Applicability of Agile Development Methods to Design for Additive Manufacturing. Procedia CIRP.

[30] Vinodh S., Devadasan S.R., Maheshkumar S., Aravindakshan M., Arumugam M., Balakrishnan K. (2010). Agile Product Development through CAD and Rapid Prototyping Technologies: An Examination in a Traditional Pump-Manufacturing Company. Int. J. Adv. Manuf. Technol..

[31] Potdar P.K., Routroy S. (2018). Analysis of Agile Manufacturing Enablers: A Case Study. Mater. Today Proc..

[32] Stare A. (2014). Agile Project Management in Product Development Projects. Procedia Soc. Behav. Sci..

[33] Raj S.A., Sudheer A., Vinodh S., Anand G. (2013). A Mathematical Model to Evaluate the Role of Agility Enablers and Criteria in a Manufacturing Environment. Int. J. Prod. Res..

[34] Chen J., Reilly R.R., Lynn G.S. (2012). New Product Development Speed: Too Much of a Good Thing?. J. Prod. Innov. Manag..

[35] Caban J., Vrabel J., Šarkan B., Zarajczyk J., Marczuk A. (2018). Analysis of the Market of Electric Tractors in Agricultural Production. MATEC Web Conf..

[36] Živanović Z., Nikolic Z. (2012). The Application of Electric Drive Technologies in City Buses. New Generation of Electric Vehicles.

[37] Lie T.T., Prasad K., Ding N. (2017). The Electric Vehicle: A Review. Int. J. Electr. Hybrid Veh..

[38] Sanguesa J.A., Torres-Sanz V., Garrido P., Martinez F.J., Marquez-Barja J.M. (2021). A Review on Electric Vehicles: Technologies and Challenges. Smart Cities.

[39] Muratori M., Alexander M., Arent D., Bazilian M., Cazzola P., Dede E.M., Farrell J., Gearhart C., Greene D., Jenn A. (2021). The Rise of Electric Vehicles—2020 Status and Future Expectations. Prog. Energy.

[40] Fishman E., Cherry C. (2016). E-Bikes in the Mainstream: Reviewing a Decade of Research. Transp. Rev..

[41] Bourne J.E., Sauchelli S., Perry R., Page A., Leary S., England C., Cooper A.R. (2018). Health Benefits of Electrically-Assisted Cycling: A Systematic Review. Int. J. Behav. Nutr. Phys. Act..

[42] Salmeron-Manzano E., Manzano-Agugliaro F. (2018). The Electric Bicycle: Worldwide Research Trends. Energies.

[43] Hung N.B., Lim O. (2020). A Review of History, Development, Design and Research of Electric Bicycles. Appl. Energy.

[44] Kazemzadeh K., Bansal P. (2021). Electric Bike Level of Service: A Review and Research Agenda. Sustain. Cities Soc..

[45] Dižo J., Blatnický M., Melnik R., Karľa M. (2022). Improvement of Steerability and Driving Safety of an Electric Three-Wheeled Vehicle by a Design Modification of Its Steering Mechanism. LOGI Sci. J. Transp. Logist..

[46] Turoń K., Czech P., Macioszek E., Sierpiński G. (2020). The Concept of Rules and Recommendations for Riding Shared and Private E-Scooters in the Road Network in the Light of Global Problems. Proceedings of the Modern Traffic Engineering in the System Approach to the Development of Traffic Networks.

[47] Gogola M. Are the E-Bikes More Dangerous than Traditional Bicycles?. Proceedings of the 2018 XI International Science-Technical Conference Automotive Safety.

[48] Tavčar J., Černe B., Duhovnik J., Zorko D. (2021). A Multicriteria Function for Polymer Gear Design Optimization. J. Comput. Des. Eng..

[49] Figlus T., Kozioł M., Kuczyński Ł. (2019). The Effect of Selected Operational Factors on the Vibroactivity of Upper Gearbox Housings Made of Composite Materials. Sensors.

[50] Singh P.K., Siddhartha, Singh A.K. (2018). An Investigation on the Thermal and Wear Behavior of Polymer Based Spur Gears. Tribol. Int..

[51] Mao K., Langlois P., Hu Z., Alharbi K., Xu X., Milson M., Li W., Hooke C.J., Chetwynd D. (2015). The Wear and Thermal Mechanical Contact Behaviour of Machine Cut Polymer Gears. Wear.

[52] Hasl C., Illenberger C., Oster P., Tobie T., Stahl K. (2018). Potential of Oil-Lubricated Cylindrical Plastic Gears. J. Adv. Mech. Des. Syst. Manuf..

[53] Hasl C., Oster P., Tobie T., Stahl K. (2017). Bending Strength of Oil-Lubricated Cylindrical Plastic Gears. Ing..

[54] Bravo A., Koffi D., Toubal L., Erchiqui F. (2015). Life and Damage Mode Modeling Applied to Plastic Gears. Eng. Fail. Anal..

[55] Trobentar B., Kulovec S., Hlebanja G., Glodež S. (2020). Experimental Failure Analysis of S-Polymer Gears. Eng. Fail. Anal..

[56] Mao K., Li W., Hooke C.J., Walton D. (2009). Friction and Wear Behaviour of Acetal and Nylon Gears. Wear.

[57] Sarita B., Senthilvelan S. (2019). Effects of Lubricant on the Surface Durability of an Injection Molded Polyamide 66 Spur Gear Paired with a Steel Gear. Tribol. Int..

[58] Lu Z., Liu H., Zhu C., Song H., Yu G. (2019). Identification of Failure Modes of a PEEK-Steel Gear Pair under Lubrication. Int. J. Fatigue.

[59] Illenberger C.M., Tobie T., Stahl K. (2019). Flank Load Carrying Capacity of Oil-Lubricated High Performance Plastic Gears. Forsch. Im. Ing..

[60] Vorgerd J., Tenberge P., Joop M. (2022). Scuffing of Cylindrical Gears with Pitch Line Velocities up to 100 m/S. Forsch. Im Ing..

[61] Ding Y., Rieger N.F. (2003). Spalling Formation Mechanism for Gears. Wear.

[62] Brumercik F., Lukac M., Caban J., Krzysiak Z., Glowacz A. (2020). Comparison of Selected Parameters of a Planetary Gearbox with Involute and Convex–Concave Teeth Flank Profiles. Appl. Sci..

[63] Wojnar G., Burdzik R., Wieczorek A.N., Konieczny Ł. (2021). Multidimensional Data Interpretation of Vibration Signals Registered in Different Locations for System Condition Monitoring of a Three-Stage Gear Transmission Operating under Difficult Conditions. Sensors.

[64] Hu J., Hu N., Yang Y., Zhang L., Shen G. (2022). Nonlinear Dynamic Modeling and Analysis of a Helicopter Planetary Gear Set for Tooth Crack Diagnosis. Measurement.

[65] Cao W., Han Z., Yang Z.Z., Wang N., Qu J.X., Wang D. (2022). Deterioration State Diagnosis and Wear Evolution Evaluation of Planetary Gearbox Using Vibration and Wear Debris Analysis. Measurement.

[66] Zhang K., Li H., Cao S., Wang C., Sun B., Liu A. (2022). Investigation on Planetary Gearbox Fault Mechanism under Variable Speed Conditions Based on Rigid-Flexible Coupling Dynamics Model. Eng. Fail. Anal..

[67] Zorko D. (2021). Investigation on the High-Cycle Tooth Bending Fatigue and Thermo-Mechanical Behavior of Polymer Gears with a Progressive Curved Path of Contact. Int. J. Fatigue.

[68] Zorko D., Kulovec S., Duhovnik J., Tavčar J. (2019). Durability and Design Parameters of a Steel/PEEK Gear Pair. Mech. Mach. Theory.

[69] Tavčar J., Grkman G., Duhovnik J. (2018). Accelerated Lifetime Testing of Reinforced Polymer Gears. J. Adv. Mech. Des. Syst. Manuf..

[70] Zorko D., Demšar I., Tavčar J. (2021). An Investigation on the Potential of Bio-Based Polymers for Use in Polymer Gear Transmissions. Polym. Test..

[71] Černe B., Petkovšek M., Duhovnik J., Tavčar J. (2020). Thermo-Mechanical Modeling of Polymer Spur Gears with Experimental Validation Using High-Speed Infrared Thermography. Mech. Mach. Theory.

[72] Cathelin J. (2019). Material Data for Advanced Plastic Gear Simulation. International Conference on Gears 2019.

[73] Zorko D., Duhovnik J., Tavčar J. (2021). Tooth Bending Strength of Gears with a Progressive Curved Path of Contact. J. Comput. Des. Eng..

[74] Zorko D., Tavčar J., Šturm R., Bergant Z. (2021). Investigation of the Durability and Performance of Autoclave-Cured, Woven Carbon Fiber-Reinforced Polymer Composite Gears in Mesh with a Steel Pinion. Compos. Struct..

[75] Zorko D., Tavčar J., Bizjak M., Šturm R., Bergant Z. (2021). High Cycle Fatigue Behaviour of Autoclave-Cured Woven Carbon Fibre-Reinforced Polymer Composite Gears. Polymer Testing.

[76] Černe B., Bergant Z., Šturm R., Tavčar J., Zorko D. (2022). Experimental and Numerical Analysis of Laminated Carbon Fibre-Reinforced Polymer Gears with Implicit Model for Coefficient-of-Friction Evaluation. J. Comput. Des. Eng..

[77] Urbas U., Zorko D., Vukašinović N., Černe B. (2022). Comprehensive Areal Geometric Quality Characterisation of Injection Moulded Thermoplastic Gears. Polymers.

